# Supplementary material for: Evaluation of Usnea barbata (L.) Weber ex F.H. Wigg Extract in Canola Oil Loaded in Bioadhesive Oral Films for Potential Applications in Oral Cavity Infections and Malignancy
Source: Antioxidants (Basel). 2022 Aug 19;11(8):1601. doi: 10.3390/antiox11081601 (PMC9404812; doi:10.3390/antiox11081601)
Supplement: Supplementary file 1 [file antioxidants-11-01601-s001.zip › Supplementary Material, Tables S1, S2, and S3.pdf]

Table S1. The inhibitory activity of F-UBO on Gram-positive (*S. aureus*) and Gram-negative bacteria (*P. aeruginosa*) after 24 ore incubation at 37 °C.

CTR (mg/mL)		*S. aureus*		*P. aeruginosa*		F-UBO
**30.230 ± 0.630**	**122.330 ± 0.850**	**CTR**	**F-UBO**	**CTR**	**F-UBO**	**63.533 ± 1.955**
1.511 ± 0.043	6.117 ± 0.042	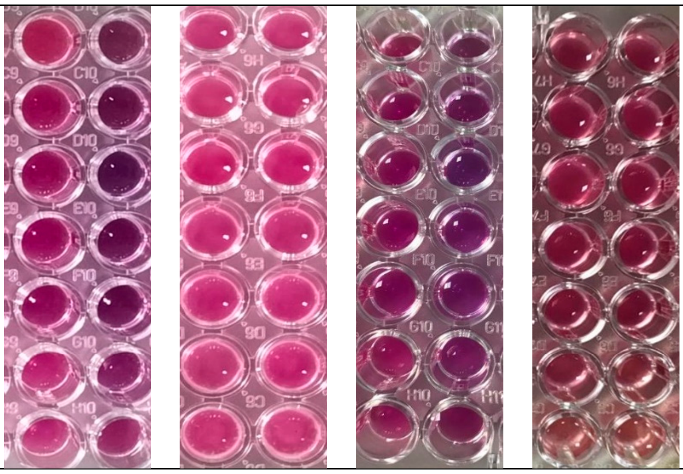				3.176 ± 0.097
0.755 ± 0.022	4.893 ± 0.034					1.588 ± 0.048
0.377 ± 0.011	3.914 ± 0.027					0.794 ± 0.024
0.188 ± 0.005	3.131 ± 0.021					0.397 ± 0.012
0.094 ± 0.002	2.505 ± 0.017					0.199 ± 0.008
0.047 ± 0.002	2.004 ± 0.014					0.100 ± 0.004
0.023 ± 0.001	1.603 ± 0.011					0.049 ± 0.002

CTR—Ceftriaxone, F-UBO—Bioadhesive oral film with *U. barbata* extract in canola oil; * results interpreted by using Resazurin dye chart, adapted from Madushan et al. [46] as follows: blue—“excellent”; light blue—"very good”; violet—“good”; purple-pink—"moderate”; light pink—“low”; pink—"very low”; white— “no effect.”

Table S2. The antibacterial and antifungal activities of 5% P407.

S. aureus		*P. aeruginosa*		*C. albicans*		*C. parapsilosis*		P407 (mg/mL)
**CTR**	**P407**	**CTR**	**P407**	**TRF**	**P407**	**TRF**	**P407**	**50.133 ± 1.305**
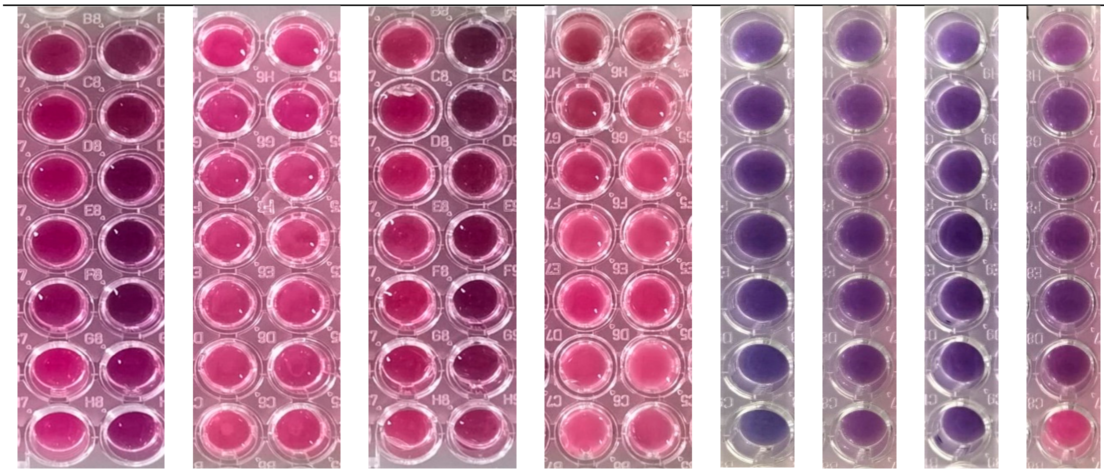								2.506 ± 0.065
								1.253 ± 0.032
								0.626 ± 0.016
								0.315 ± 0.008
								0.157 ± 0.004
								0.078 ± 0.002
								0.039 ± 0.001
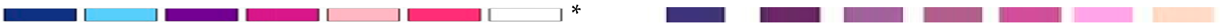 *							**	
**(a)**	**(b)**	**(c)**	**(d)**	**(e)**	**(f)**	**(g)**	**(h)**	

CTR—Ceftriaxone, P407—Poloxamer 407, TRF—Terbinafine
* Results interpreted by using Resazurin dye chart, adapted from Madushan et al. [46] as follows: blue—“excellent”; light blue—"very good”; violet—“good”; purple-pink—"moderate”; light pink—“low”; pink—"very low”; white— “no effect” (a-d)
** Results interpreting adapted from Bitacura et al. [48] (e-h)

Table S3. The inhibitory activity of F-UBO on *C. albicans* and *C. parapsylosis* after 24 ore incubation at 35 °C; the color score and signification [48].

**Dil.**	**TRF (mg/mL)** 10.050 ± 0.180	***C. albicans*** TRF	F-UBO	***C. parapsilosis*** TRF	F-UBO	**Color** **	**Score** **	**Signification****
1	0.500 ± 0.009	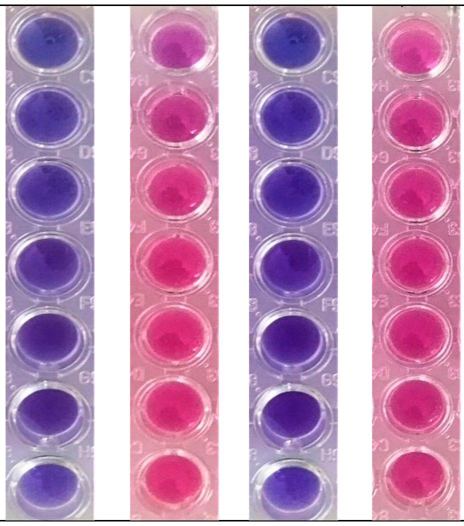					0	Blue – cells are death
2	0.250 ± 0.004						1	Violet-blue – cells are partially death
3	0.125 ± 0.002						2	Violet – cells are alive; no proliferation
4	0.061 ± 0.001						3	Light-violet – low proliferation
5	0.031 ± 0.001						4	Dark pink – moderate proliferation
6	0.015 ± 0.001						5	Pink – fast proliferation
7	0.007 ± 0.001						6	Light pink – very fast proliferation

TRF—Terbinafine, F-UBO—bioadhesive films with *U. barbata* extract in canola oil.
** Results interpreting adapted from Bitacura et al. [48].
